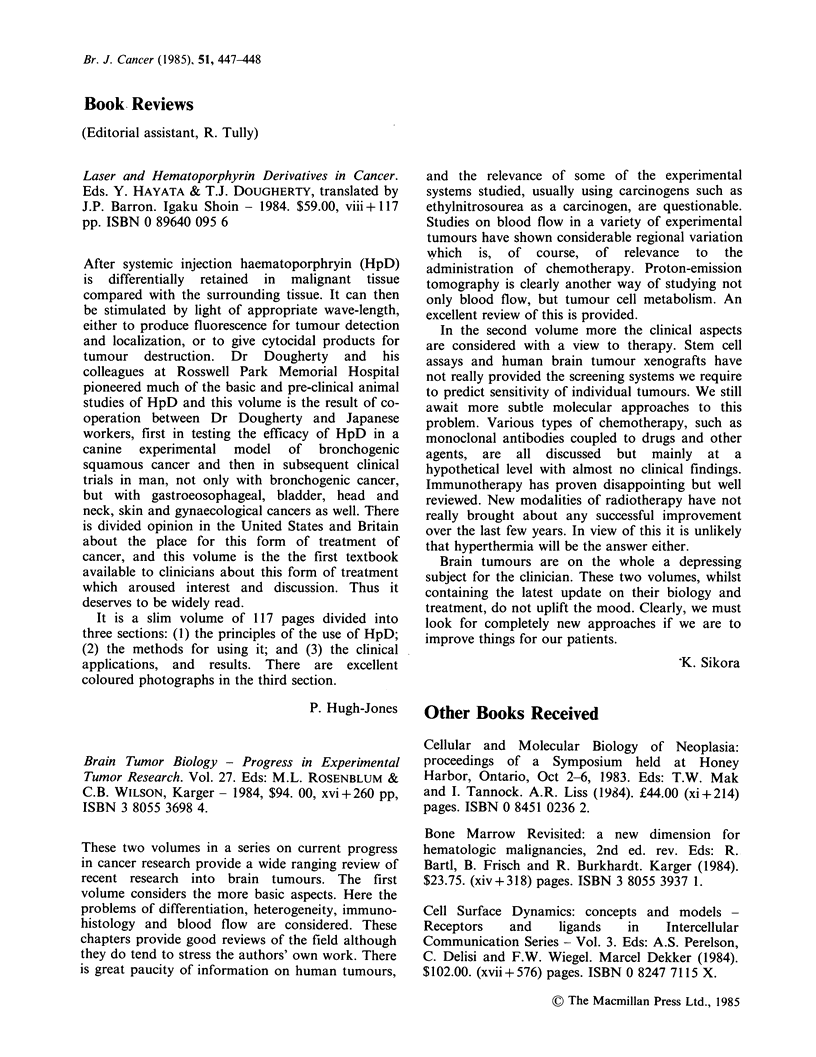# Brain Tumor Biology - Progress in Experimental Tumor Research

**Published:** 1985-03

**Authors:** K. Sikora


*Brain Tumor Biology – Progress in Experimental Tumor Research.* Vol. 27. Eds: M.L. ROSENBLUM & C.B. WILSON, Karger – 1984, $94. 00, xvi + 260 pp, ISBN 3 8055 3698 4.

These two volumes in a series on current progress in cancer research provide a wide ranging review of recent research into brain tumours. The first volume considers the more basic aspects. Here the problems of differentiation, heterogeneity, immuno-histology and blood flow are considered. These chapters provide good reviews of the field although they do tend to stress the authors' own work. There is great paucity of information on human tumours, and the relevance of some of the experimental systems studied, usually using carcinogens such as ethylnitrosourea as a carcinogen, are questionable. Studies on blood flow in a variety of experimental tumours have shown considerable regional variation which is, of course, of relevance to the administration of chemotherapy. Proton-emission tomography is clearly another way of studying not only blood flow, but tumour cell metabolism. An excellent review of this is provided.

In the second volume more the clinical aspects are considered with a view to therapy. Stem cell assays and human brain tumour xenografts have not really provided the screening systems we require to predict sensitivity of individual tumours. We still await more subtle molecular approaches to this problem. Various types of chemotherapy, such as monoclonal antibodies coupled to drugs and other agents, are all discussed but mainly at a hypothetical level with almost no clinical findings. Immunotherapy has proven disappointing but well reviewed. New modalities of radiotherapy have not really brought about any successful improvement over the last few years. In view of this it is unlikely that hyperthermia will be the answer either.

Brain tumours are on the whole a depressing subject for the clinician. These two volumes, whilst containing the latest update on their biology and treatment, do not uplift the mood. Clearly, we must look for completely new approaches if we are to improve things for our patients.

K. Sikora